# Factors affecting early and late period outcomes in patients undergoing surgical repair due to biliary tract injury

**DOI:** 10.3389/fsurg.2025.1576454

**Published:** 2025-07-03

**Authors:** İbrahim Çoğal, Burak Yavuz, Yunus Kaycı, Uğur Topal, İshak Aydın, Ahmet Gokhan Saritas, Kubilay Dalci, İsmail Cem Eray, Atilgan Tolga Akcam, Abdullah Ülkü

**Affiliations:** Department of General Surgery, Cukurova University Faculty of Medicine, Adana, Türkiye

**Keywords:** biliary tract injuries, hepaticojejunostomy, cholecystectomy, patient care team, treatment outcome

## Abstract

**Aim:**

Cholecystectomy is the most common elective abdominal surgery globally. With the advent of laparoscopy, laparoscopic cholecystectomy has become the gold standard. However, this has also led to an increase in biliary tract injuries, a complication with high morbidity and mortality that requires a multidisciplinary treatment approach. This study aims to identify factors influencing postoperative outcomes in patients undergoing surgical repair for biliary tract injuries.

**Materials and method:**

This study included 66 patients referred to the General Surgery Department of Çukurova University Medical Faculty for biliary tract injuries between January 2005 and June 2022, all of whom underwent hepaticojejunostomy. Demographic data, pre- and post-operative lab values, imaging, and anastomosis types were recorded and analyzed. Early and long-term postoperative outcomes were examined, using the McDonald classification for long-term follow-up.

**Results:**

Of the 66 patients, 18 (27.3%) were male, and 48 (72.7%) were female, with a mean follow-up of 105 ± 58 months. Early postoperative complications developed in 28 patients (42.4%). Diabetes and culture positivity were significantly associated with wound infections. Elevated pre- and post-repair ALP and GGT levels were significantly associated with poorer long-term outcomes according to the McDonald classification. Vascular injury was significantly associated with isolated ALP-GGT elevation. Anastomotic stricture developed in 8 patients (12.1%). Of these, 2 (3%) were successfully managed with balloon dilation

**Conclusion:**

Biliary tract injury is a serious complication post-cholecystectomy, requiring a multidisciplinary approach and follow-up in a hepatobiliary center. Surgeon experience and local risk factors are crucial in managing these injuries.

## Introduction

Biliary tract injuries are one of the most serious complications that can occur during cholecystectomy, the most commonly performed elective abdominal surgery worldwide. With the advancement of laparoscopy, laparoscopic cholecystectomy has become the gold standard treatment method; however, the risk of biliary tract injuries has increased 2–3 times compared to open cholecystectomy (1–3).

These injuries can lead to complications such as bile leakage, biloma, recurrent cholangitis, secondary biliary cirrhosis, liver failure, and in severe cases, death (4).

When a biliary tract injury occurs, the patient's treatment should be performed in an advanced center equipped with a surgical team experienced in hepatobiliary surgery, gastroenterology, and interventional radiology (5, 6).

In patients undergoing surgical repair, early postoperative complications such as wound infection, anastomotic leakage, and cholangitis are frequently observed, prolonging hospital stay and recovery. Studies report that up to 42.9% of patients experience at least one postoperative complication, including surgical site infections (7.4%), cholangitis (5.7%), anastomotic leak (4.6%), intraabdominal abscess, and biloma (3.4%). In the long term, patients may require rehospitalization due to recurrent cholangitis, pain, or anastomotic strictures, which typically develop within the first two years but can emerge even a decade after surgery. Given the high risk of both early and late complications, lifelong follow-up remains essential, even after a seemingly successful surgical repair (7). Therefore, even after a successful surgical repair, considering the frequency of early postoperative complications and long-term complications, lifelong follow-up is necessary.

In this study, we aim to present the factors affecting postoperative early and late period outcomes in patients who underwent surgical repair due to biliary tract injuries, in light of the literature.

## Materials and method

The study was conducted at the Department of General Surgery, Çukurova University Medical Faculty, and involved patients who underwent surgical repair due to biliary tract injuries between January 2005 and June 2022. Ethical approval for this research was granted by Çukurova University Faculty of Medicine Non-Interventional Clinical Research Ethics Committee, on September 1, 2023, under decision number 136/23. Patient data were accessed through the hospital's file archive unit and electronic information management system. The study included 66 patients.

Patients included in the study were those who developed biliary tract injuries following cholecystectomy and required surgical repair. All surgical procedures were performed by the same hepatobiliary surgical team, eliminating variability in surgeon experience. Patients treated with endoscopic or percutaneous interventions, as well as those who were operated on due to malignancy and developed biliary injury during the procedure, were excluded from the study. All cases were referrals from external centers. Antibiotic therapy was adjusted based on culture results, and patients were treated with appropriate wound care. The demographic data of all patients, their symptoms at the time of referral, clinical, laboratory, and radiological findings, type of injury, time elapsed until definitive surgery, surgical procedure undertaken, early postoperative findings, and long-term outcomes during outpatient follow-ups were examined. The presence of any concurrent vascular injuries prior to or during definitive surgery and the type of incision used were also reviewed.

Bile duct injuries were classified according to the Strasberg classification system, which categorizes injuries based on anatomical location and severity. Type A includes bile leaks from the cystic duct or small ducts in the liver bed. Type B refers to occlusion of an aberrant right hepatic duct without bile leakage. Type C involves transection of an aberrant right hepatic duct with bile leakage. Type D represents partial transection of the common bile duct. Type E injuries, further subdivided into E1–E5, indicate strictures or complete transections of the common bile duct at various levels. Only patients with Strasberg Type E injuries were included in the research, as less severe injuries (Types A–D) are often managed non-surgically, primarily through endoscopic or percutaneous interventions by gastroenterology teams.

Early postoperative outcomes were assessed for complications. Postoperative complications were classified as biloma, bile fistula, jaundice, peritonitis, pleural effusion, urinary tract infection, wound site infection, cholangitis, other complications, or no complications. Factors affecting these outcomes were examined, and statistical analysis was conducted.

Patients were followed up in the first month postoperatively and then every three months. Follow-up intervals after the first year were extended based on the patients' conditions. During outpatient visits, the presence of symptoms, laboratory values, and radiological imaging were recorded. Long-term follow-up outcomes included the absence of symptoms, cholangitis, anastomotic stricture, isolated ALP-GGT elevation, signs of chronic liver disease, necessity for percutaneous dilation or anastomotic revision surgery.

The McDonald classification was used to evaluate late-term treatment outcomes (8). According to this classification, the patients were grouped as follows: asymptomatic patients with normal liver function tests were classified as McDonald A, asymptomatic patients with isolated liver enzyme elevation as McDonald B, patients with symptoms requiring hospitalization (e.g., pain, cholangitis, fever) as McDonald C, and patients with biliary strictures requiring endoscopic or percutaneous intervention as McDonald D.

### Statistical analysis

IBM SPSS v22.0 was used for the statistical analysis of the data. The normality of numerical data was assessed using the Shapiro–Wilk test. Numerical data following a normal distribution were presented as mean ± standard deviation, while non-normally distributed numerical data were presented as median (interquartile range). Categorical data were presented as frequencies and percentages. For comparisons between groups, normally distributed numerical data were analyzed using the *T*-test, while non-normally distributed numerical data were analyzed using the Mann–Whitney *U*-test. The chi-square test was used for comparisons of categorical variables between groups. A *p*-value of less than 0.05 was considered statistically significant.

## Results

Our study included 66 patients, with 18 males (27.3%) and 48 females (72.7%). The average age of the patients was 46.8 ± 13.9 (range 21–76 years). Biliary tract injuries occurred post-cholecystectomy in 15 patients (22.7%) after open cholecystectomy and in 51 patients (77.3%) following laparoscopic cholecystectomy. In 10 patients, conversion from laparoscopy to open surgery was performed. 7 patients had attempted repair before being referred to us, but it was unsuccessful.

Upon referral to our clinic, the symptoms observed at admission included abdominal pain in 48 patients, bile fistula in 32, jaundice in 29, and vomiting in 17. The symptoms are detailed in Table 1.

**Table 1:** Patients’ symptoms at the time of admission to the clinic following referral.

Complaint	*n*/*N*	%
Jaundice	29/66	43.9%
Fistula	32/66	48.5%
Abdominal Pain	48/66	72.7%
Fever	14/66	21.2%
Vomiting	17/66	25.8%
Peritonitis	9/66	13.6%
Chills	5/66	7.6%
Itching	3/66	4.5%

The time elapsed post-biliary tract injury until surgical repair was categorized into four groups. Twenty-seven patients (40.9%) underwent repair within 4–7 days, 16 (24.2%) within 1–3 days, 8 within 8–10 days, and 15 after more than 10 days. However, the timing of the repair had no significant effect on early or late complications.

Laboratory findings at the time of admission are shown in Table 2.

**Table 2:** Labaratory results of patients.

Laboratory findings	*N*	%
Amylase (U/L)		
Normal	45	68.2
60–100	8	12.1
Over 100	13	19.7
INR		
Normal	52	78.8
Between 1,2–2	12	18.2
Over 2	2	3
ALT (U/L)		
0–50	23	34.8
51–100	17	25.8
101–500	24	36.4
Over 500	2	3
AST (U/L)		
0–50	27	40.9
51–100	15	22.7
101–500	24	36.4
Direct Bilirubin (mg/dl)		
Normal	33	50
2–5	22	33.3
5–10	8	12.1
10–15	2	3
Over 15	1	1.5
WBC (10^3^/µl)		
Normal	33	50
10–15,000	22	33.3
Over 15,000	11	16.7
Parameters:	Median(interquartile range)	
ALP (U/L)	194 (132–384)	
GGT (U/L)	154 (84–248)	
CRP (mg/L)	11 (7–27)	

INR, international normalized ratio; ALT, alanine transaminase; AST, aspartate transaminase; ALP, alkaline phosphatase; GGT, gamma glutamyl transferase; CRP, C- reactive protein; WBC, White blood count.

Vascular injuries were present in 10 patients (15.2%), all of whom had right hepatic artery injuries.

The distribution of biliary tract injury levels according to classifications is presented in Table 3. Hepaticojejunostomy was performed in 78.8% (*n* = 52) of the cases, portoenterostomy in 15.2% (*n* = 10), and cholangiojejunostomy in 6.1% (*n* = 4).

**Table 3:** Distribution of patients according to biliary tract injury classifications.

Strasberg type	*n*	%
Strasberg		
E1	41	62.1
E2	18	27.3
E3	4	6.1
E4	2	3.0
E5	1	1.5

Early and long-term outcomes of the patients are shown in Table 4.

**Table 4:** Postoperative complications—early and late period outcomes.

Postoperative complications	*N*	%
Early complications		
Biliary Fistula	1	1.5
Bilioma	3	4.5
Pleural effusion	14	
Urinary Tract Infection	4	6.1
Surgical Site Infection	16	24.2
Pneumonia	6	9.1
Cholangitis	2	3
Other	6	9.1
Late complications		
Cholangitis	10	15.2
Anastomotic Stenosis	8	12.1
Isolated ALP-GGT elevation	13	19.7
Chronic Liver Disease	1	1.5
Liver Mass	2	3.0
Asymptomatic	39	59.1
Incisional Hernia	6	9.1

ALP, alkaline phosphatase; GGT, gamma glutamyl transferase.

The average follow-up period was 105 ± 58 (1–216) months. Patient follow-up findings were evaluated using the McDonald score: Grade A in 44 patients, Grade B in 8, Grade C in 5, and Grade D in 8 patients.

Factors affecting early postoperative complications were evaluated. The presence of diabetes and positive culture results were significantly associated with the development of wound site infections (*p:* 0.032, *p:* 0.001). No significant relationship was found between patient age, symptom duration, incision type, and laboratory findings on the development of postoperative complications (*p* *>* 0.05).

The McDonald classification was used in long-term follow-ups, with a long-term morbidity rate of 19.6% (McDonald C-D). Anastomotic stricture developed in 8 patients during follow-ups. Two patients responded to balloon dilation, while revision surgery was performed in 5 patients who did not respond to balloon dilation. One patient, who failed balloon dilation at 12 months postoperatively, was planned for revision surgery but expired due to cholangitic sepsis. The time until stricture development post-biliary tract repair was 19.8 (12–36) months. Revision surgeries were conducted at 13, 20, 24, 66, and 134 months postoperatively.

In evaluating factors affecting long-term follow-up outcomes, we found that pre-repair ALP levels significantly influenced the McDonald classification, creating a meaningful difference between McDonald A and D groups (*p:*0.018). However, pre-repair GGT and CRP levels did not significantly affect the McDonald classification (*p* *>* 0.05).

A significant relationship was found between ALP and GGT levels measured within the first three months post-repair and the McDonald classification. There was a significant relationship between ALP levels and McDonald A–C groups (*p:*0.04) and McDonald A–D groups (*p* *<* 0.001). A significant relationship was also found between GGT levels and all McDonald classification groups (*p* *<* 0.05), as shown in Table 5.

**Table 5:** Relationship between McDonald groups based on laboratory values.

Laboratory values	McDonald A–B	McDonald A–C	McDonald A–D
Pre-operative ALP	*p*: 1.000	*p*: 0.454	***p*: 0.018**
Postoperative ALP	*p*: 0.066	***p*: 0.04**	***p* *<* 0.001**
Postoperative GGT	***p*: 0.014**	***p*: 0.002**	***p*: 0.001**

Bold indicate significant values *p* < 0.05.

ALP, alkaline phosphatase; GGT, gamma glutamyl transferase.

The presence of vascular injury was significantly related to the McDonald score (*p:*0.02) and was associated with elevated ALP-GGT levels during follow-ups (*p* *<* 0.05). Among the 10 patients with vascular injuries, 5 experienced isolated ALP-GGT elevation, 2 developed anastomotic strictures, 1 had recurrent cholangitis attacks, while 2 remained asymptomatic.

## Discussion

Biliary tract injury is a rare but life-threatening complication following cholecystectomy. The incidence of biliary tract injury post-laparoscopic cholecystectomy is around 0.5%–0.9% (2). Although the incidence of biliary tract injuries has decreased with increased experience, the rate continues to persist (1, 3).

In this study, we aimed to present the factors affecting early and late-term outcomes of patients who were referred to us from external centers due to biliary tract injury (Strasberg type E) and underwent hepaticojejunostomy. A total of 66 patients were included in the study. The average age of the patients was 46.8 ± 13.9 years (min: 21, max: 76). The average follow-up duration of the patients was 105 ± 58 months (12–216 months).

When evaluating early postoperative outcomes, 16 patients had wound infections, 14 had pleural effusions, 6 had lung infections, 4 had urinary tract infections, 3 had bilomas, and 3 had gastrointestinal bleeding. The presence of diabetes and culture positivity were identified as risk factors for wound infection development (*p*: 0.032) (*p*: 0.001). Age, symptom duration, incision type, and laboratory findings were found to have no effect on early postoperative outcomes.

Late-term follow-up findings were evaluated using the McDonald classification. 44 patients were classified as McDonald A, 8 as McDonald B, 5 as McDonald C, and 8 as McDonald D. The long-term morbidity rate was determined to be 19.6% (McDonald C-D). Preoperative ALP (*p*: 0.012) and postoperative ALP-GGT (*p* < 0.05) were found to affect the McDonald classification, while preoperative GGT and CRP (*p*: 0.091) (*p*: 0.903) did not. Additionally, the presence of associated vascular injury was found to affect the McDonald classification (*p* < 0.05) and to have a statistically significant relationship with isolated elevated ALP-GGT in follow-ups (*p* < 0.05). Other laboratory findings, injury level, anastomosis level, and the presence of preoperative SIRS did not affect long-term outcomes. Anastomotic strictures were detected in 8 patients. The mean time to the development of anastomotic stricture was determined to be 19.8 (12–36) months.

There are few studies sharing early period outcomes in patients who underwent surgical repair due to biliary tract injury. In one study that shared early period outcomes, it was reported that 42.9% of patients experienced one or more postoperative complications. The complications reported included wound site infection (7.4%), cholangitis (5.7%), anastomotic leak (4.6%), intraabdominal abscess, and biloma (3.4%) (8). In our study, 42.4% (*n*:28) of patients experienced one or more postoperative complications. There were wound site infections in 16 patients, pleural effusion in 14, urinary tract infection in 4, biloma in 3, cholangitis in 2, and gastrointestinal bleeding in 3 patients. Only one patient expired due to MI in the first postoperative month. No other postoperative mortality occurred.

Studies report that while postoperative mortality within the first 30 days is around 2%, the mortality seen in the first 5 years is reported as 20% (9). In our study, one patient experienced postoperative mortality. Apart from this patient, 7 others expired due to reasons unrelated to biliary tract pathology. One patient expired due to cholangitic sepsis after an unsuccessful attempt at balloon dilation for anastomotic stricture at 12 months postoperatively.

Biomarkers such as CRP, procalcitonin, and serum lactate levels can be used to assess the severity of inflammation and response to treatment, and may indicate progression to poor outcomes and mortality (10, 11). In our study, no significant relationship was found between CRP levels and postoperative complications. We determined that preoperative CRP levels had no significant effect on McDonald scores in post-repair follow-ups (*p:*0.903). We believe this is due to the early initiation of treatment and repair in patients with biliary tract injuries before they become septic.

There is ongoing debate regarding the timing of surgical repair. Various studies have shown that delayed surgical repair has a lower risk of postoperative complications compared to early repair (1, 4, 12–14). However, there are publications advocating for early repair (15–18) and studies showing similar short and long-term outcomes for early and delayed repairs (6, 19–21). The aim of delayed repair is to control the septic condition, restore vascular injuries, and stabilize the patient's clinical condition. It also allows for the final outcome of biliary canal ischemia to be determined, establishing the level of anastomosis. On the other hand, early repair can prevent further deterioration of the patient's clinical condition and lead to shorter hospital stays and lower costs (22). In our study, the timing of the repair was not found to have a significant impact on early or late complications. The timing of the repair is generally assessed on a case-by-case basis, considering the patient's clinical condition.

High-level biliary canal anastomosis is recommended to prevent anastomotic leaks due to ischemia (23). In our study, hepaticojejunostomy was performed in 78.8% (*n*:52) of patients, portoenterostomy in 15.2% (*n*:10), and cholangiojejunostomy in 6.1% (*n*:4). Biloma developed in 3 patients and bile fistula in 1 patient, who had undergone portoenterostomy. One patient underwent percutaneous drainage catheter placement, while others were followed up with drains until complete regression of biloma and spontaneous closure of the bile fistula.

Routine blood tests are not highly recommended following laparoscopic cholecystectomy due to the possibility of mild elevation in cholestasis enzymes. However, biochemical parameters should be considered after difficult cases or when clinical findings suggest complications (24–26). Direct and indirect bilirubin, AST, ALT, ALP, GGT, and WBC are generally used to detect iatrogenic biliary tract injuries (24, 25). Liver function tests and cholestatic enzymes can be elevated or remain within normal ranges in biliary tract injuries. Bilirubin levels rise in cases of stenosis in the biliary tract, but laboratory values can remain normal in cases of bile leakage.

There is no study in the literature investigating the effect of pre-repair ALP-GGT and CRP levels on post-repair follow-up complication development in cases of biliary tract injury. In our study, we found that pre-repair ALP levels had a significant effect on the McDonald classification used in postoperative follow-ups (*p:*0.012). However, GGT and CRP levels did not significantly affect the McDonald classification (*p:*0.091, *p:* 0.903). We believe that patients with high pre-repair ALP levels should be closely monitored postoperatively as they may be at risk of developing cholangitis or anastomotic stricture (McDonald C-D). We also found that ALP-GGT elevation in the first three months postoperatively had a significant effect on the McDonald classification (*p* *<* 0.05). High or non-decreasing ALP-GGT levels during follow-ups may indicate the development of cholangitis or anastomotic stricture in the long term.

Current studies indicate that vascular injuries occurring alongside biliary tract injuries usually happen during the transition from laparoscopic to open surgery (27, 28). The presence of vascular injury in biliary tract injuries can lead to unsuccessful repair outcomes or long-term strictures (17, 18, 29). In our study, we found that the presence of vascular injury had a significant effect on McDonald classification in follow-ups (*p:*0.02) and was significantly associated with post-repair ALP-GGT elevation (*p* *<* 0.05). Of the 10 patients with vascular injuries, 5 experienced isolated ALP-GGT elevation, 2 developed anastomotic strictures and underwent balloon dilation, 1 had a history of hospitalization due to postoperative cholangitis, and 2 were asymptomatic.

In the literature, the rate of anastomotic stricture development varies between 4.1% and 69%, but most studies report a rate of 10%–20%. The average time for the development of anastomotic stricture is reported as 11–30 months. The presence of vascular injury, level of injury, presence of sepsis or peritonitis, and post-repair bile leak are associated with poor outcomes (30). In our study, we found that vascular injury had a significant effect on the McDonald classification in postoperative follow-ups among the 10 patients with vascular injuries. According to the Strasberg classification, 41 patients (62.1%) had E1, 18 (27.3%) E2, 4 E3, 2 E4, and 1 E5 injury. No significant difference was found in postoperative complications and McDonald classification based on injury type (*p* *>* 0.05).

In patients with stricture development in our study, PTC with balloon dilation was initially attempted, and revision surgery was performed in those who did not benefit. Revision surgeries were carried out at 13, 20, 24, 66, and 134 months postoperatively. Therefore, long-term follow-up is necessary in patients who have experienced biliary tract injuries. In our clinic, we conducted follow-ups every three months starting from the first postoperative month. After the first year, follow-ups were conducted annually.

Our findings highlight the importance of individualized patient management and long-term follow-up in cases of biliary tract injury. Given the significant impact of vascular injury and elevated ALP-GGT levels on postoperative outcomes, early identification of high-risk patients is crucial. A structured follow-up protocol incorporating biochemical markers and imaging may help in the early detection of complications such as anastomotic stricture and cholangitis. Additionally, optimizing surgical techniques and perioperative care in specialized hepatobiliary centers can contribute to improved long-term outcomes, reinforcing the necessity of a multidisciplinary approach in the management of these complex cases.

Our study has several limitations. First, it is a retrospective analysis conducted on patients referred from external centers, which may introduce selection bias. Additionally, the inclusion of only Strasberg Type E injuries may limit the generalizability of the findings, as these represent more complex referral cases. Data on intraoperative blood loss and operative time were not available, as these parameters were not routinely recorded in the hospital's electronic medical record system during the early years of the study period. The lack of homogeneous distribution of anastomosis levels also prevented meaningful comparisons based on anastomosis type. Future prospective studies with larger cohorts and standardized follow-up protocols are needed to validate these findings.

## Conclusion

Biliary tract injury following cholecystectomy leads to significant morbidity and mortality, necessitating prolonged follow-up after surgical repair. It is important to emphasize that the three-month postoperative marker was selected as a practical early surveillance point; persistent elevation of ALP and GGT beyond this period may serve as an early warning sign for the development of anastomotic stricture, rather than an immediate diagnostic criterion. Knowing the factors associated with complications will guide us toward personalized treatment in high-risk patients. Early referral of patients with suspected or confirmed injuries to an experienced center will improve outcomes.

## Data Availability

The raw data supporting the conclusions of this article will be made available by the authors, without undue reservation.
